# Supplementary material for: Haemolysis Detection in MicroRNA-Seq from Clinical Plasma Samples
Source: Genes (Basel). 2022 Jul 21;13(7):1288. doi: 10.3390/genes13071288 (PMC9317737; doi:10.3390/genes13071288)
Supplement: Supplementary file 1 [file genes-13-01288-s001.zip › Figure S1.pdf]

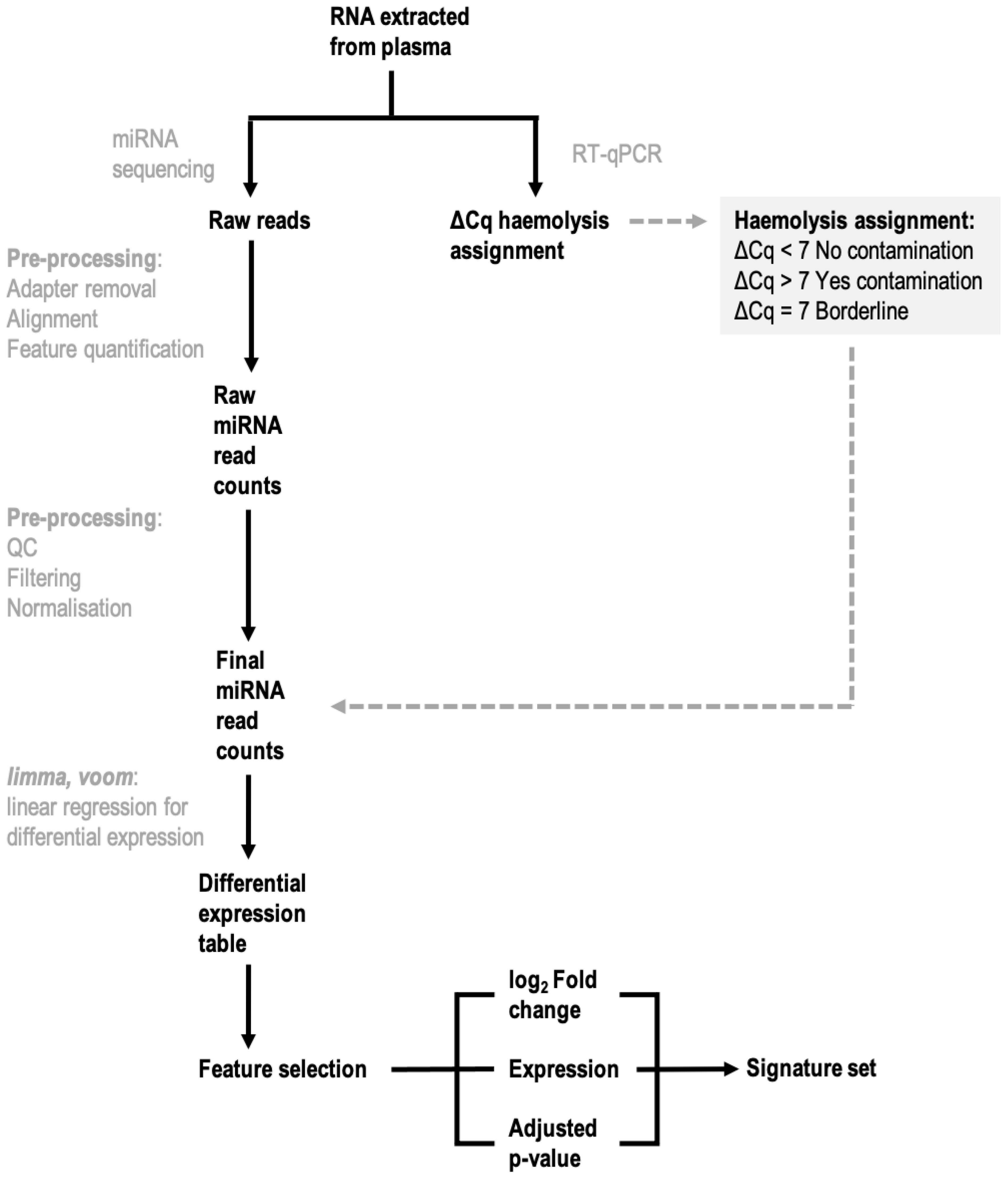

Supplementary figure S1: Haemolysis signature feature selection. Raw single-end reads from small RNA-seq libraries are pre-processed using a range of Unix- and python-based computational tools to quantify miRNA expression in each library. Data quality is ensured through quality control steps throughout the workflow. Concurrently with sequencing, ΔCq (miR-23a-miR-451) was assessed by RT-qPCR and incorporated into the differential expression analysis.
