# Supplementary material for: Haemolysis Detection in MicroRNA-Seq from Clinical Plasma Samples
Source: Genes (Basel). 2022 Jul 21;13(7):1288. doi: 10.3390/genes13071288 (PMC9317737; doi:10.3390/genes13071288)
Supplement: Supplementary file 1 [file genes-13-01288-s001.zip › Figure S2.pdf]

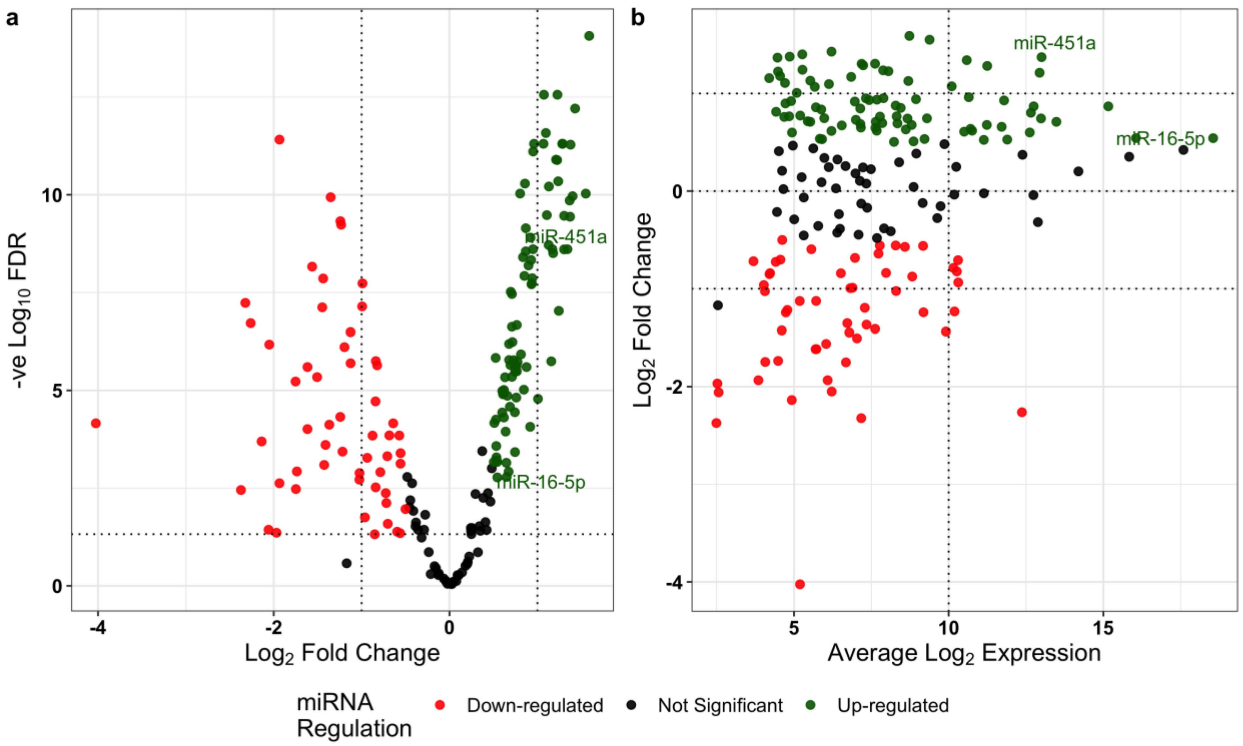

Supplementary Figure S2: (a) Volcano plot of differential expression. Linear regression identified 138 miRNA which were more highly abundant in haemolysed compared to non-haemolysed samples with FDR < 0.05 (green). (b) MA plot (M (log ratio) and A (mean average)) of Log_2 fold change as a function of Log_2 average expression indicates most miRNA have an average expression < 10 Log_2 CPM. miR-451a and miR-16-5p, both highly red blood cell associated, are highly expressed and more abundant in the haemolysed group (green).
