# Supplementary material for: Haemolysis Detection in MicroRNA-Seq from Clinical Plasma Samples
Source: Genes (Basel). 2022 Jul 21;13(7):1288. doi: 10.3390/genes13071288 (PMC9317737; doi:10.3390/genes13071288)
Supplement: Supplementary file 1 [file genes-13-01288-s001.zip › Figure S3.pdf]

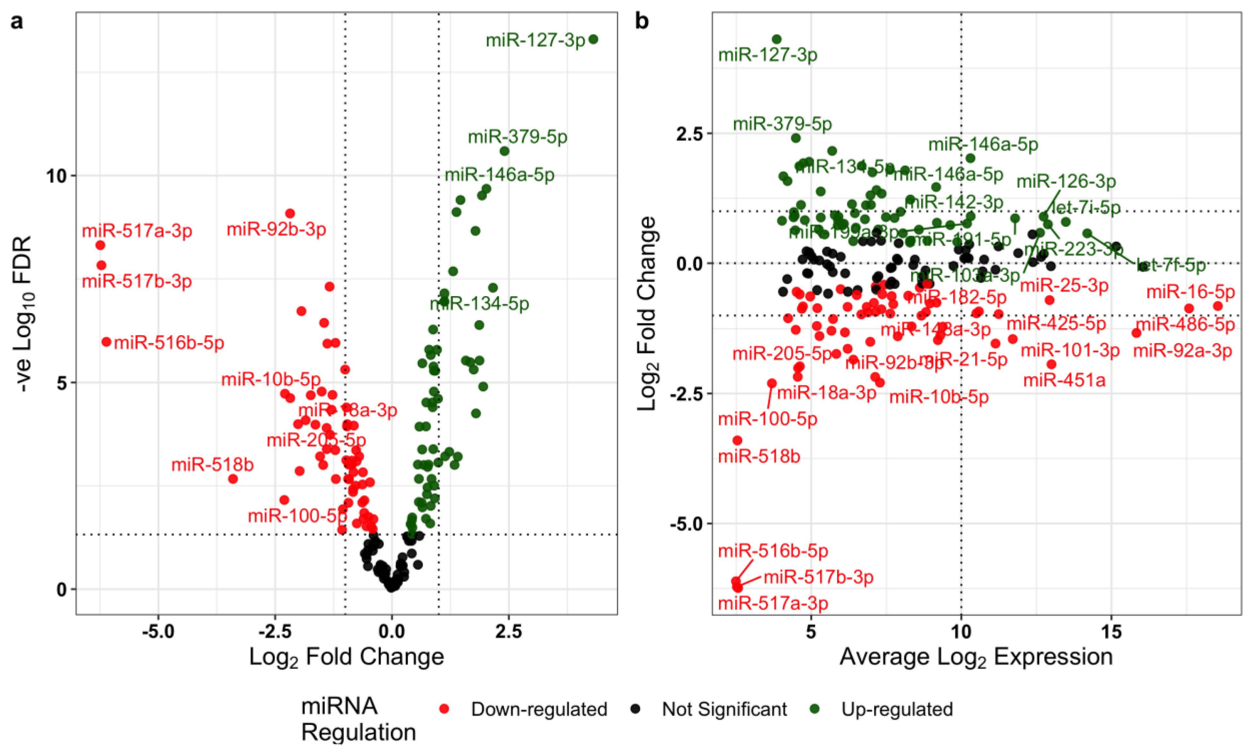

Supplementary Figure S3: (a) Volcano plot of differential expression between the pregnant and non-pregnant samples. Linear regression identified 104 miRNA (FDR < 0.05) which were more highly abundant in the pregnant population (red). Haemolysis Metric signature miRNAs are labelled (b) MA plot (M (log ratio) and A (mean average)) of Log_2 fold change as a function of Log_2 average expression indicates most miRNA have an average expression < 10 Log_2 CPM. Unsurprisingly, the most differentially expressed miRNA are miR-517a-3p, miR-517b-3p, miR-516b-5p, miR-518b, which are all members of the highly placenta associated chromosome 19 miRNA cluster.
