# Supplementary material for: Haemolysis Detection in MicroRNA-Seq from Clinical Plasma Samples
Source: Genes (Basel). 2022 Jul 21;13(7):1288. doi: 10.3390/genes13071288 (PMC9317737; doi:10.3390/genes13071288)
Supplement: Supplementary file 1 [file genes-13-01288-s001.zip › Figure S4.pdf]

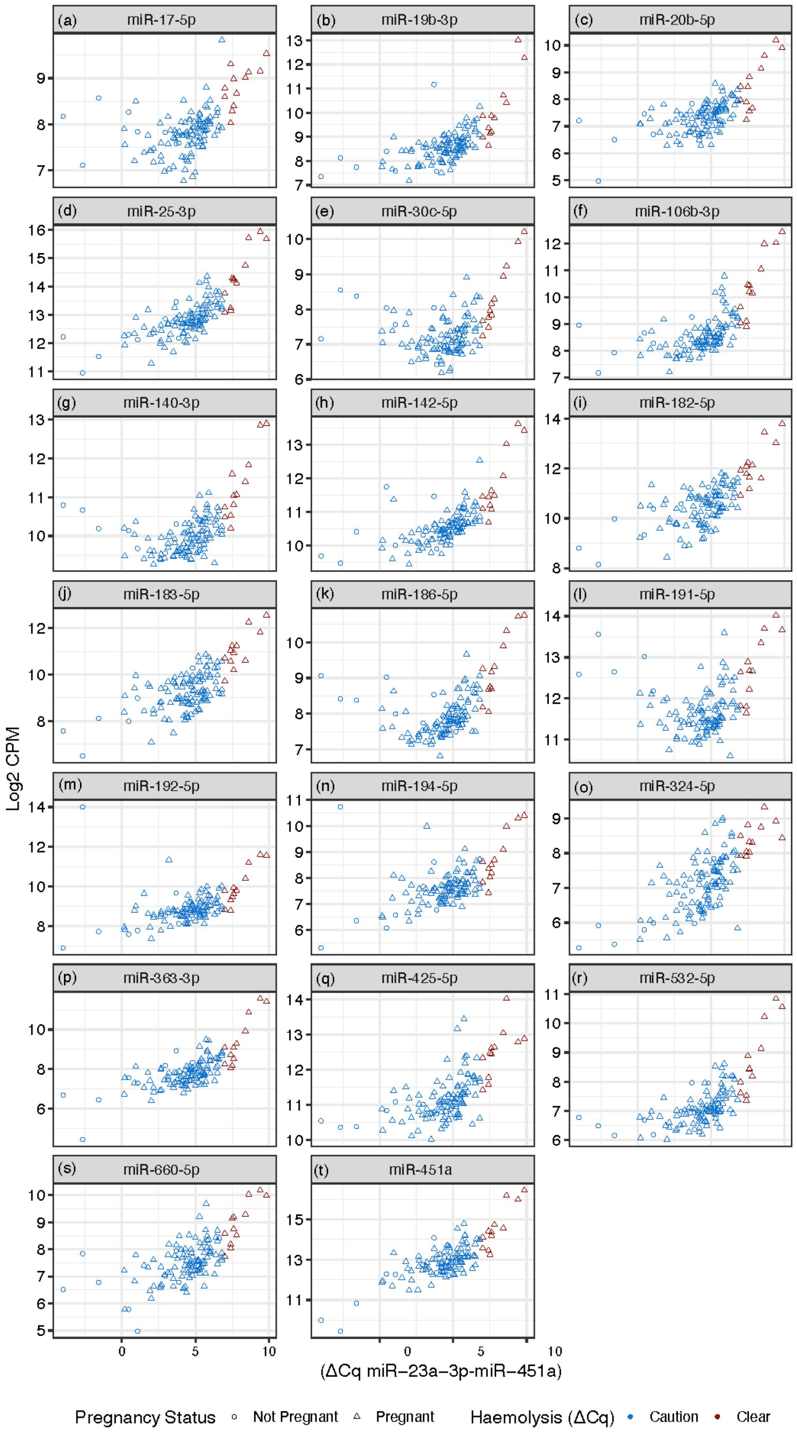

Supplementary Figure S4.1: Scatter plots of Log_2 expression for all miRNAs in our 20 miRNA haemolysis signature set as a function of the ΔCq (miR-451a-miR-23a-3p) gold standard method of haemolysis detection. Signature miRNAs are presented in ascending numeric order from miR-17-5p (a) to miR-451a (t). For each miRNA there is a strong positive correlation between expression and the sample measure of ΔCq (miR-451a-miR-23a-3p).

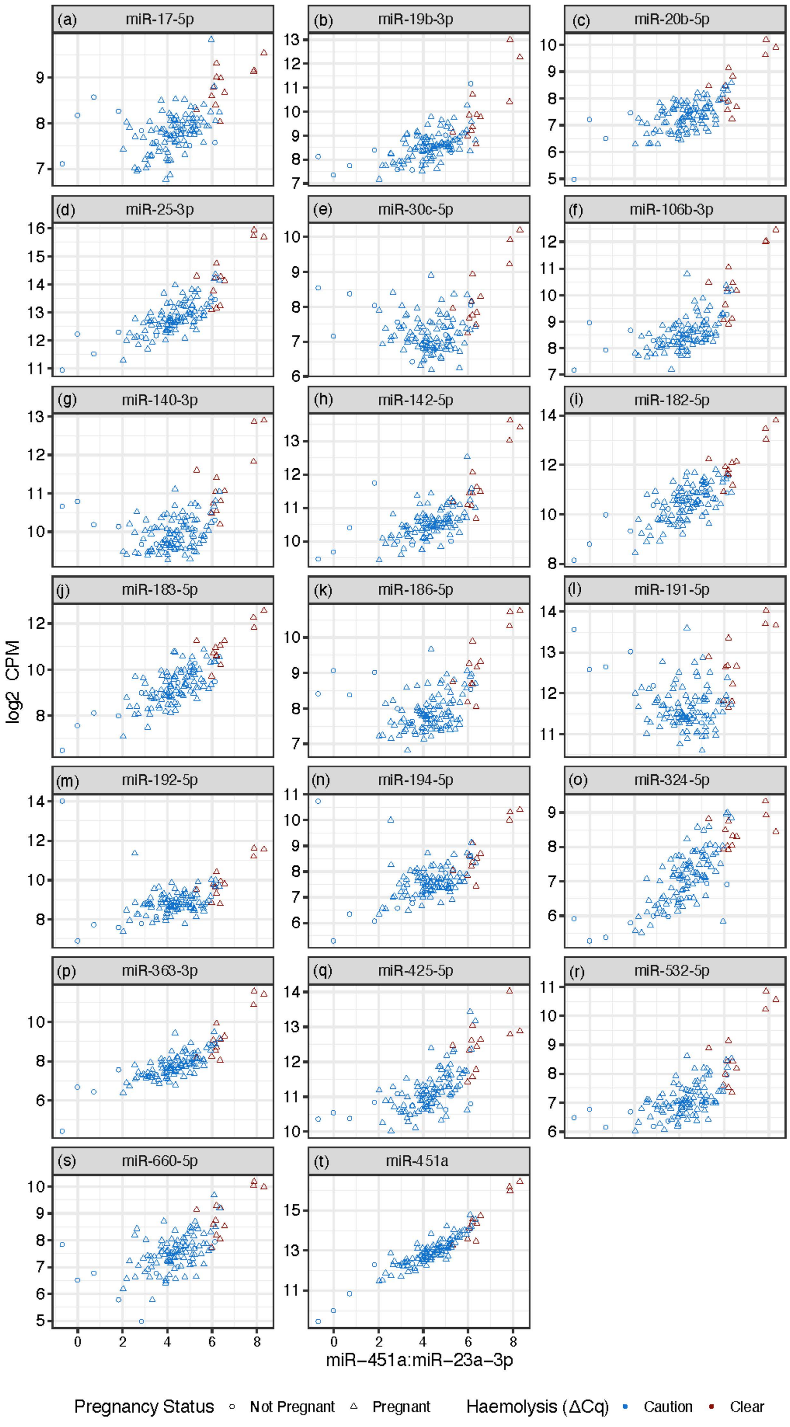

Supplementary Figure S4.2: Scatter plots of log_2 signature microRNA (miRNA) counts per million (CPM) as a function of a simple, proxy, measure of haemolysis calculated by subtracting the log_2 CPM expression of the invariant miRNA miR-23a-3p from the red blood cell associated miR-451a. Samples are coloured according to the measure of a haemolysis assayed using the ΔCq (miR-451a-miR-23a-3p) gold standard method. For all signature miRNAs there is a strong correlation between the expression of the signature miRNAs and the gold standard haemolysis metric.

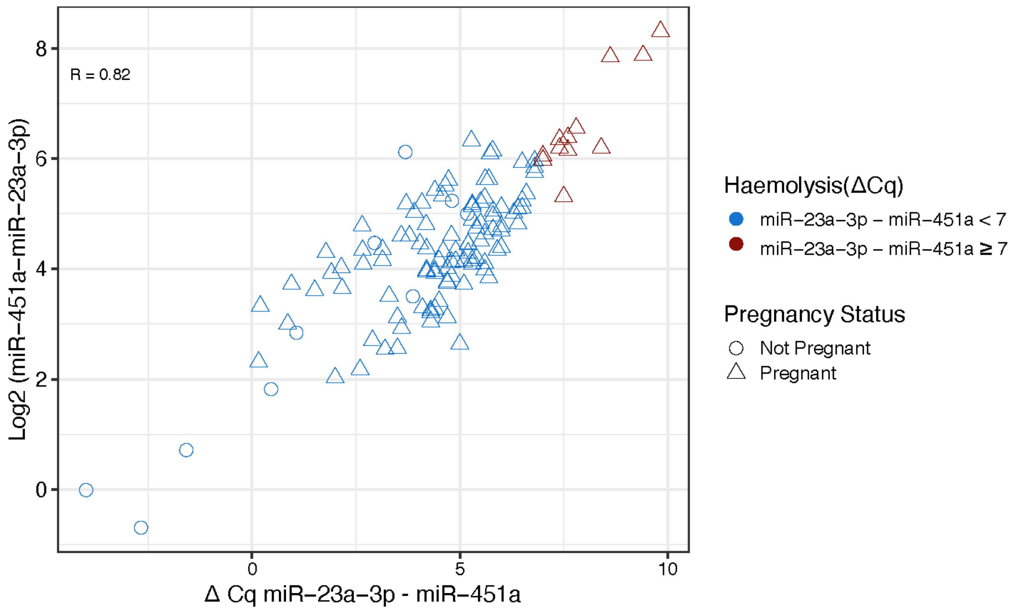

Supplementary Figure S4.3: Scatter plot of the simple, proxy, measure of haemolysis calculated by subtracting the $\log_2$ CPM expression of the invariant miRNA miR-23a-3p from the red blood cell associated miR-451a as a function of the ΔCq (miR-451a-miR-23a-3p) gold standard method of haemolysis detection. We identified a strong, statistically significant (P-Value < 2.2×10^{-16}) correlation between these two measures of haemolysis.
