# Supplementary material for: Haemolysis Detection in MicroRNA-Seq from Clinical Plasma Samples
Source: Genes (Basel). 2022 Jul 21;13(7):1288. doi: 10.3390/genes13071288 (PMC9317737; doi:10.3390/genes13071288)
Supplement: Supplementary file 1 [file genes-13-01288-s001.zip › Figure S5.pdf]

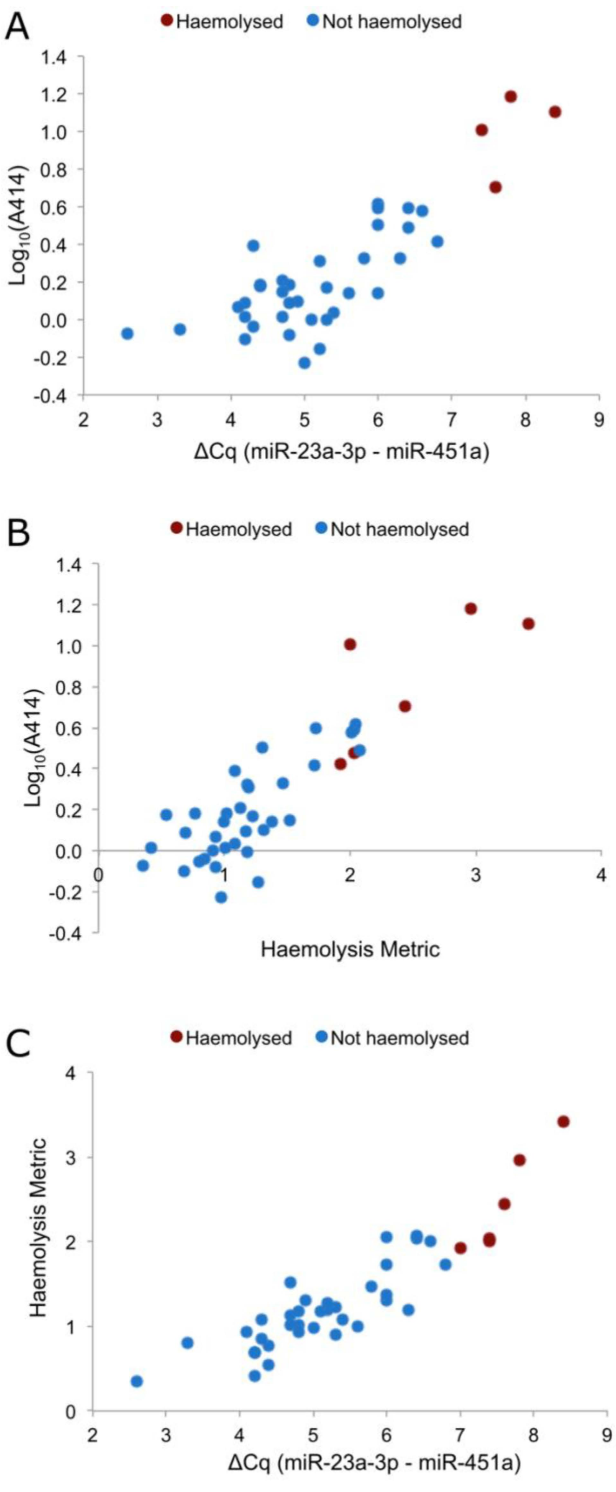

Supplementary Figure S5. Comparison of the Spectrophotometry Absorbance:ΔCq RT-qPCR (A), Spectrophotometry Absorbance:derived Haemolysis Metric (B), and derived Haemolysis Metric:ΔCq RT-qPCR (C) methods for estimating haemolysis. This was performed for a subset of 41 samples for which we had data from all three methods of haemolysis evaluation. The samples are colored according to the haemolysis classification from the ΔCq method. A clear correlation is present between all three methods and Haemolysis Metric described in this work was more highly correlated with both the Absorbance (R = 0.87) and ΔCq (R=0.90) methods than they are with each other (R=0.82).
