# Supplementary material for: Haemolysis Detection in MicroRNA-Seq from Clinical Plasma Samples
Source: Genes (Basel). 2022 Jul 21;13(7):1288. doi: 10.3390/genes13071288 (PMC9317737; doi:10.3390/genes13071288)
Supplement: Supplementary file 1 [file genes-13-01288-s001.zip › Figure S6.pdf]

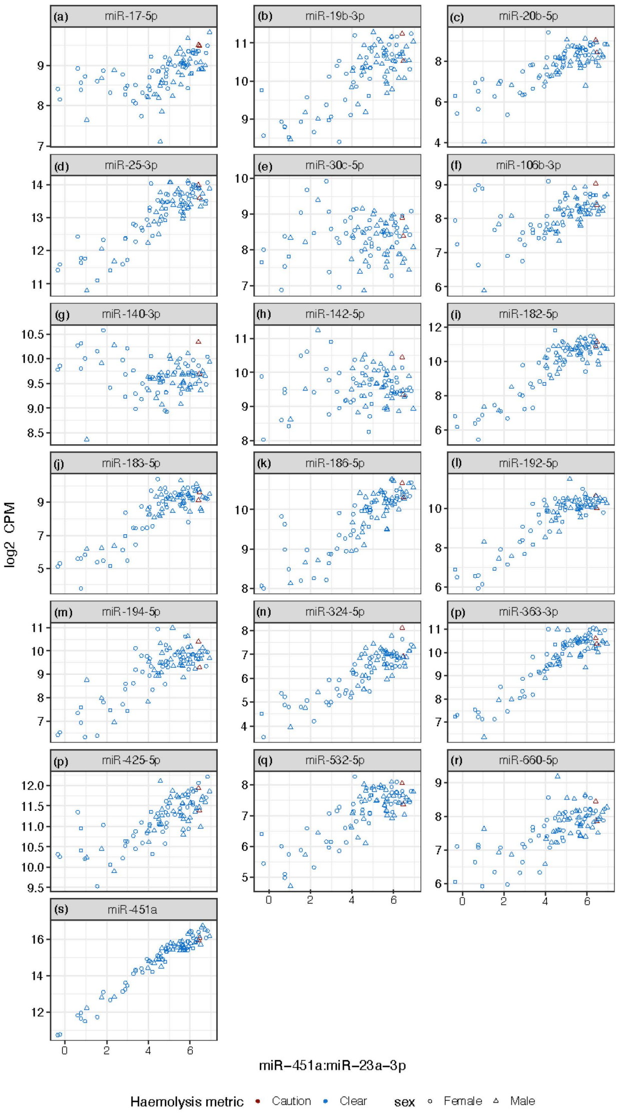

Supplementary Figure S6.1: Public dataset GSE151341 (radiographic knee osteoarthritis). Scatter plots of $\log_2$ signature microRNA (miRNA) counts per million (CPM) as a function of a simple, proxy, measure of haemolysis calculated by subtracting the $\log_2$ CPM expression of the invariant miRNA miR-23a-3p from the red blood cell associated miR-451a. Signature miRNAs are presented in ascending numeric order from miR-17-5p (a) to miR-451a (s). Importantly, in this mixed experiment containing both male and female patients (n=91) we see no clustering of haemolysis signature miRNA expression by sex. We found a strong correlation between our haemolysis signature miRNAs and the proxy measure of haemolysis. There were no signature miRNAs associated with this data reported dysregulated in radiographic knee arthritis (12). Notably, miR-191-5p is negatively correlated with the proxy measure of haemolysis. Whilst this study did not identify miR-191-5p as dysregulated in radiographic knee osteoarthritis, miR-191-3p was identified as dysregulated in their Patient:Control experiment. Previous studies have shown miR-191-5p to be upregulated in patients with hand osteoarthritis (38), highlighting the need for careful consideration of confounding signals when calculating the Haemolysis metric.

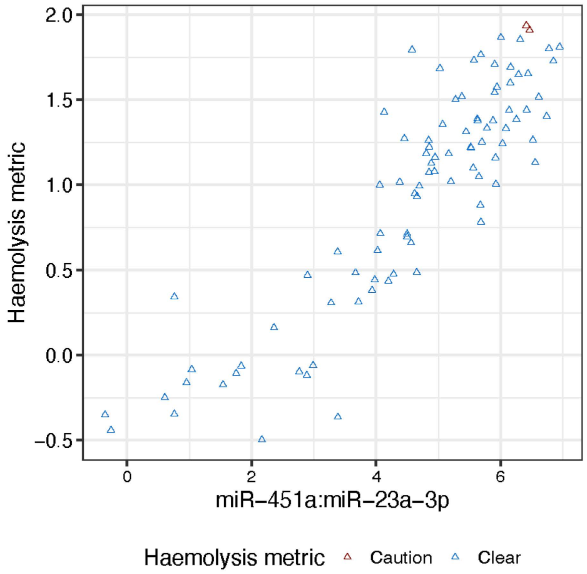

Supplementary Figure S6.2: Public dataset GSE151341 (radiographic knee osteoarthritis). Scatter plot of the Haemolysis metric as a function of the proxy measure of haemolysis calculated by subtracting the log_2 CPM expression of the invariant miRNA miR-23a-3p from the red blood cell associated miR-451a. Both *in silico* measures of haemolysis show a strong correlation.

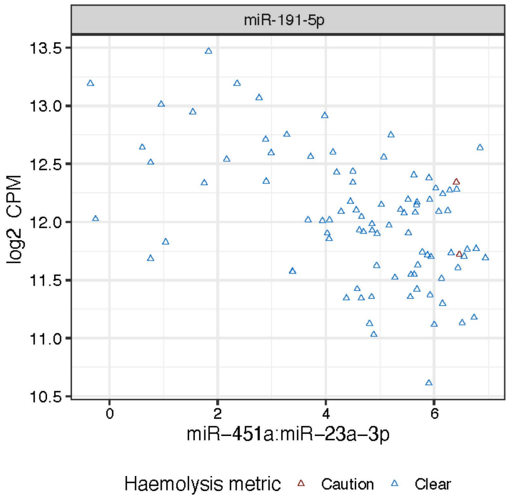

Supplementary Figure S6.3: Public dataset GSE151341 (radiographic knee osteoarthritis). Scatter plot of miR-191-5p log_2 CPM expression as function of the proxy haemolysis metric calculated by subtracting the log_2 CPM expression of the invariant miRNA miR-23a-3p from the red blood cell associated miR-451a. miR-191-5p is the signature miRNA dropped from calculation of the Haemolysis metric in accordance with our recommendations. As anticipated, miR-191-5p does not have a strong positive correlation with the proxy measure of haemolysis.
