# Supplementary material for: Haemolysis Detection in MicroRNA-Seq from Clinical Plasma Samples
Source: Genes (Basel). 2022 Jul 21;13(7):1288. doi: 10.3390/genes13071288 (PMC9317737; doi:10.3390/genes13071288)
Supplement: Supplementary file 1 [file genes-13-01288-s001.zip › Figure S7.pdf]

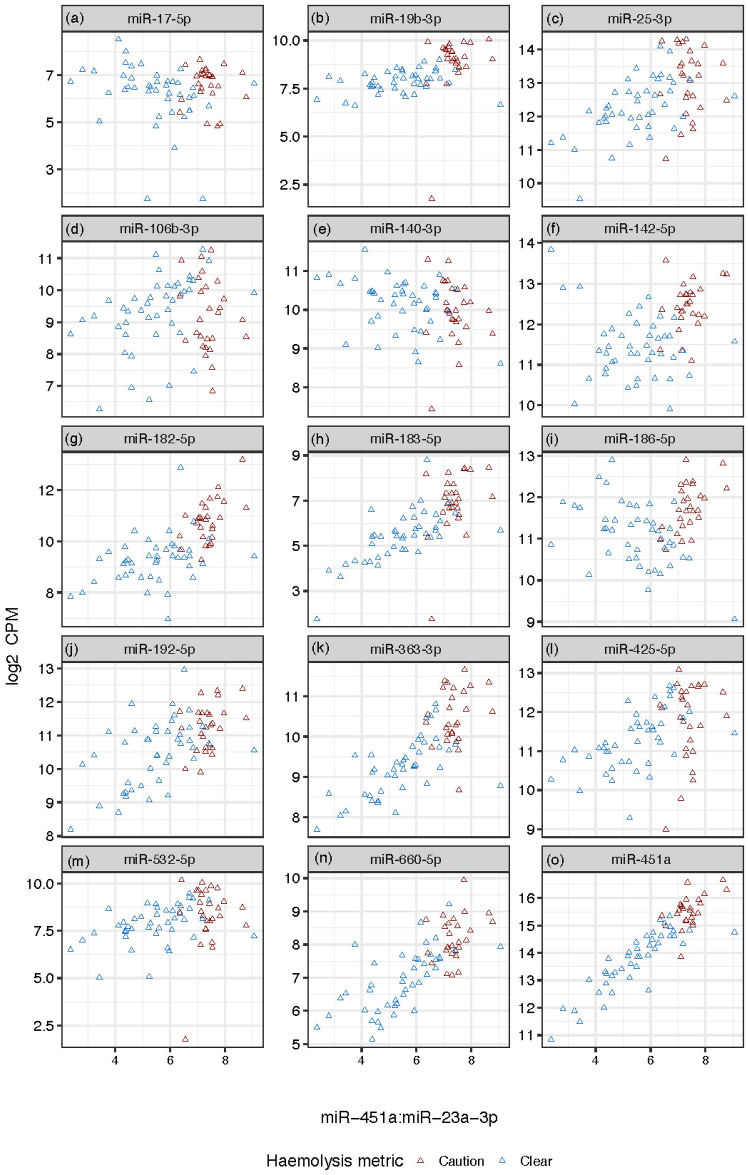

Supplementary Figure S7.1: Public dataset GSE118038 (prostate cancer study). Scatter plots of $\log_2$ signature microRNA (miRNA) counts per million (CPM) as a function of a simple, proxy measure of haemolysis calculated by subtracting the $\log_2$ CPM expression of the invariant miRNA miR-23a-3p from the red blood cell associated miR-451a. Signature miRNAs used in the calculation of the Haemolysis metric are presented in ascending numeric order from miR-17-5p (a) to miR-451a (o). Importantly, in this all-male experiment (n=70), the haemolysis signature miRNAs correlate strongly with the proxy measure of haemolysis. The publication associated with this data reported dysregulation of miR-30c-5p in prostate cancer (37). A further literature search identified miR-191-5p as dysregulated in prostate cancer with a previous study reporting this miRNA to be upregulated in patients with prostate cancer. In accordance with our method, this miRNA was excluded, as were miR-324-5p, miR-194-5p and miR-20b-5p that were found to have no expression profile in this dataset.

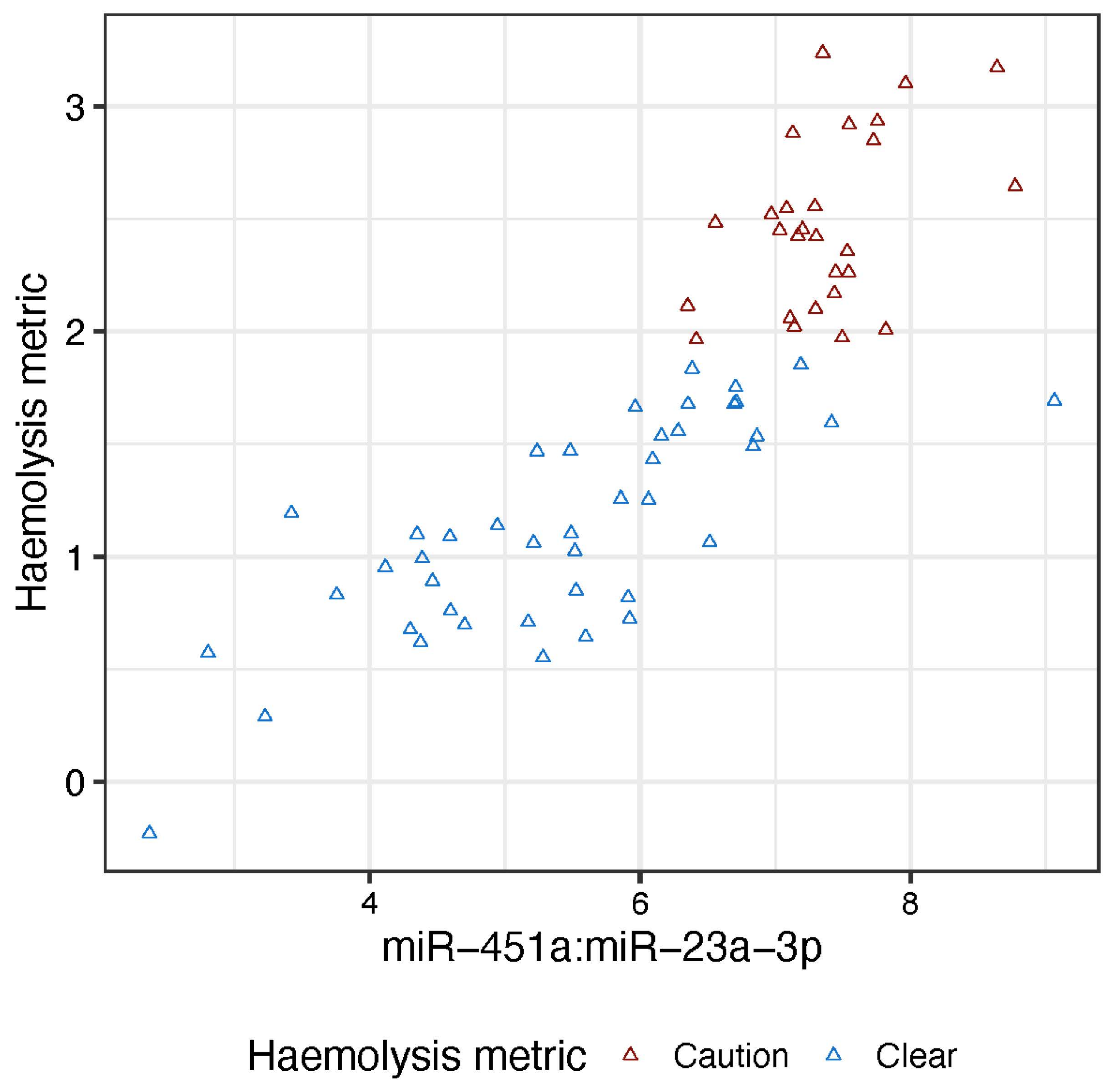

Supplementary Figure S 7.2: Public dataset GSE118038 (prostate cancer study). Scatter plot of the Haemolysis metric as a function of the proxy measure of haemolysis calculated by subtracting the log_2 CPM expression of the invariant miRNA miR-23a-3p from the red blood cell associated miR-451a. Both *in silico* measures of haemolysis show a strong correlation.

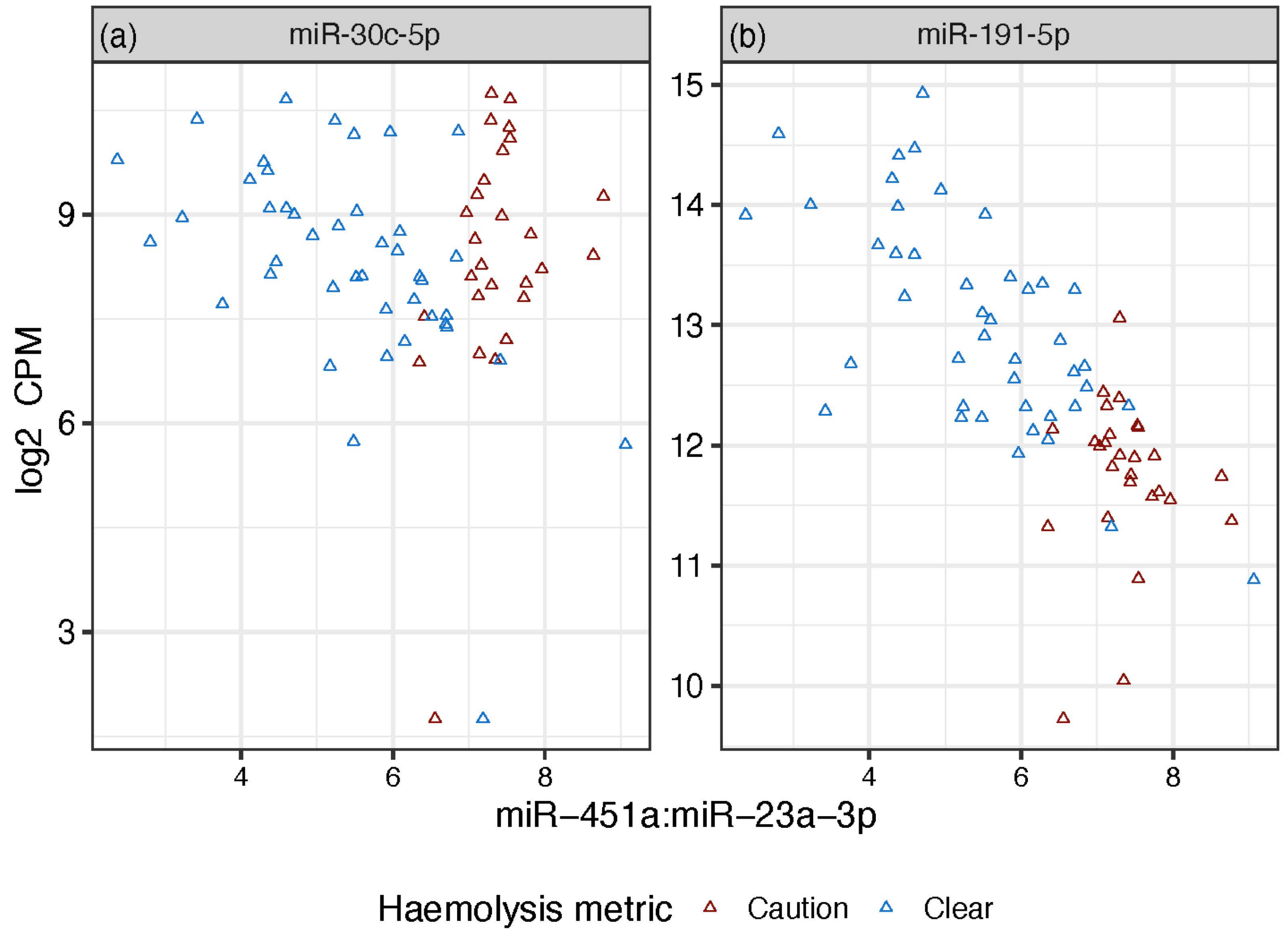

Supplementary Figure S7.3: Public dataset GSE118038 (prostate cancer study). Scatter plots of the two signature miRNAs dropped from calculation of the Haemolysis metric in accordance with our recommendations. As anticipated, neither miR-30c-5p (a) or miR-191-5p (b) have a strong positive correlation with the proxy measure of haemolysis calculated by subtracting the log_2 CPM expression of the invariant miRNA miR-23a-3p from the red blood cell associated miR-451a.
